# Multi-Objective Optimization of Friction Stir Welding Process Parameters of AA6061-T6 and AA7075-T6 Using a Biogeography Based Optimization Algorithm

**DOI:** 10.3390/ma10050533

**Published:** 2017-05-15

**Authors:** Mehran Tamjidy, B. T. Hang Tuah Baharudin, Shahla Paslar, Khamirul Amin Matori, Shamsuddin Sulaiman, Firouz Fadaeifard

**Affiliations:** 1 Department of Mechanical and Manufacturing Engineering, University Putra Malaysia, 43400 Serdang, Selangor, Malaysia; mehrantamjidy@gmail.com (M.T.); shamsuddin@upm.edu.my (S.S.); 2 Department of Industrial Engineering, Bandar Abbas Branch, Islamic Azad University, 79158 Bandar Abbas, Iran; shahlapaslar@gmail.com; 3 Institute of Advanced Technology, University Putra Malaysia, 43400 Serdang, Selangor, Malaysia; khamirul@upm.edu.my; 4 Materials Synthesis and Characterization Laboratory, Institute of Advanced Technology, Universiti Putra Malaysia, 43400 Serdang, Malaysia; f.fadaeifard@yahoo.com; 5 Advanced Manufacturing Research Centre, University Putra Malaysia, 43400 Serdang, Selangor, Malaysia

**Keywords:** friction stir welding (FSW), multi-objective biogeography based optimization (MOBBO), mathematical regression model, decision making technique

## Abstract

The development of Friction Stir Welding (FSW) has provided an alternative approach for producing high-quality welds, in a fast and reliable manner. This study focuses on the mechanical properties of the dissimilar friction stir welding of AA6061-T6 and AA7075-T6 aluminum alloys. The FSW process parameters such as tool rotational speed, tool traverse speed, tilt angle, and tool offset influence the mechanical properties of the friction stir welded joints significantly. A mathematical regression model is developed to determine the empirical relationship between the FSW process parameters and mechanical properties, and the results are validated. In order to obtain the optimal values of process parameters that simultaneously optimize the ultimate tensile strength, elongation, and minimum hardness in the heat affected zone (HAZ), a metaheuristic, multi objective algorithm based on biogeography based optimization is proposed. The Pareto optimal frontiers for triple and dual objective functions are obtained and the best optimal solution is selected through using two different decision making techniques, technique for order of preference by similarity to ideal solution (TOPSIS) and Shannon’s entropy.

## 1. Introduction

Friction stir welding (FSW) was invented at The Welding Institute (TWI) and has been successfully employed to weld aluminum alloys, particularly 2XXX or 7XXX series aluminum alloys, which are especially difficult to weld by using fusion welding techniques [1]. FSW is an attractive welding technique due to its low cost, having reduced weight by joining light-weight metals with metallurgical properties, and a reduced need for human skill [2]. In addition, its energy efficiency and environmental friendliness makes FSW a “green” process [3]. By using this welding technique, it has been reported that it is possible to reduce the consumption of energy by 99% and installation cost by 40% in comparison with resistance spot welding [4]. On the other hand, FSW is a solid-state, hot-shear joining process in which a non-consumable rotational tool is used to produce frictional heat at the welding location without material melting [5].

Researchers and practitioners are welding many different combinations of dissimilar alloys and materials due to their requirements in varied service conditions [6,7]. AA6XXX and AA7XXX Al alloys are two series of the most widely used structural materials in the automotive, rail transportation, and aerospace industries [8]. Alloy 6061 Al is the most used of the 6XXX series aluminum alloys and possesses superior weldability as compared to other heat treatable alloys and is the most popular aluminum alloy extrusion [9]. The medium strength (Al-Mg-Si) aluminum alloys such as AA6061 are extremely suited for applications in marine structures, pipelines, and storage tanks [7]. One of the strongest aluminum alloys used in today’s manufacturing industry is AA7075 (Al-Zn-Mg-Cu). AA7075 has high strength compared to its weight, combined with its natural ageing characteristics, which makes it attractive for aerospace structural applications [10].

It is well know that for any joining method, the main challenge for many manufacturers is to select the welding process parameters that will create a high quality welded joint [6,7]. It is clear that much progress has been made in FSW of dissimilar materials, but it has to be understood that study on this topic is far from enough and is still in the feasibility stages [8]. Also, what is often not considered or achieved are optimized welding process parameter combinations for FSW dissimilar aluminum alloys [11]. Since FSW involves complex interactions between many parameters that contribute in the process, the development of proper physical models for FSW which can anticipate the process’s main characteristics is a crucial concern. Thus, researchers attract development of the models based on empirical models. In this respect, response surface methodology (RSM) is a practical method of analysing, improving, and optimizing the structures over the feasible domain of parameter settings. This method is a collection of statistical and mathematical techniques in which a response of interest is influenced by several variables. RSM reduces the number of experimental trials needed to evaluate multiple parameters and their interactions [12]. Thus, it is less substantial without consuming time, material, and labour efforts [11,13]. Rajakumar and Balasubramanian [14] employed RSM to develop empirical relationships relating the FSW input parameters such as rotational speed, traverse speed, axial force, shoulder diameter, pin diameter, and tool hardness for three outputs; tensile strength, hardness, and corrosion rate of the FSW joints of the AA1100 alloy. Periyasamy et al. [15] used RSM to establish the relationship between the FSW process parameters (tool rotational speed, traverse speed, and axial force) and the responses (ultimate tensile strength (*UTS*), notch tensile strength, and weld nugget hardness) of FSW of cast AA6061 with 20% $SiC_{p}$. Ashok Kumar and Murugan [16] developed two regression models using RSM in order to predict the UTS and *E*% of the friction stir welded AA6061/$AIN_{p}$ composite by correlating the process parameters including the tool rotational speed, traverse speed, axial force, and percentage of reinforcement. Rambabu et al. [17] employed RSM to develop a mathematical model to predict the corrosion resistance of the FSW AA2219 aluminum alloy by incorporating FSW process parameters. Kadaganchi et al. [18] utilized the response surface method to develop a regression model to predict the responses of mechanical properties of friction stir welds of the AA 2014-T6 Al alloy such as ultimate tensile strength, yield strength, and percentage elongation (*E*%). Venkateswarlu et al. [19] developed a mathematical model using RSM regression analysis to predict the effects of tool geometry and process variables (i.e., rotational speed, axial force, and traverse speed) on the dissimilar FSW of the AA2219 and AA7039 alloys joint.

It is obvious that welding processes have multiple responses. In order to optimize a process with multiple objectives, various multi-objective optimization approaches such as statistical techniques and evolutionary algorithms provide good results. In the case of statistical techniques, Kasman [20] combined Taguchi with grey relational analysis to optimize a multi-response FSW of AA6082 and AA5754 Al alloys. Rajakumar and Balasubramanian [14] employed the desirability approach to find the optimal conditions in order to maximize UST and minimize the corrosion rate for FSW AA1100 Al alloys. In the case of metaheuristic algorithms, Teimouri and Baseri [13] developed a fuzzy network with an artificial bee colony and imperialist competitive algorithm for both forward and backward mapping of friction stir welded aluminum joints in order to maximize tensile strength, elongation, and hardness. Roshan et al. [21] employed adaptive neuro-fuzzy inference systems to determine the relationship between the main factors of the process such as tool pin profile, tool rotary speed, welding speed, and axial force, and the main responses including tensile strength, yield strength, and hardness of FSW aluminum 7075 plates. Then, the developed models were applied as an objective function to find the optimal process parameters by using a simulated annealing algorithm. Shojaeefard et al. [10] performed a study to model and Pareto optimize the mechanical properties of friction stir welded AA7075/AA5083. To this aim, they developed an artificial neural network model to simulate the relationship between the UTS and hardness of butt joints of AA7075-AA5083 as a function of rotational and welding speeds. Moreover, a multi-objective particle swarm optimization was employed to obtain the Pareto optimal solutions. Finally, to determine the best compromise solution, the TOPSIS approach was applied. Furthermore, Naghibi et al. [22] carried out an experimental investigation for modelling and parametric optimization of the FSW process to maximize UST and elongation for dissimilar FSW joints of AA5052 and AISI 304. In order to determine the correlation between process parameters such as tool rotational speed, welding speed, tool offset, and the mentioned responses, a back-propagation neural network was designed and developed. The developed model was then associated with the genetic algorithm to find the optimal process parameters.

One of the main contributions of this study is to solve the addressed problem by applying an efficient metaheuristic algorithm. In 2008, a new population-based evolutionary algorithm based on the geographic distribution of biological organisms was firstly introduced by Simon [23], titled biogeography based optimization (BBO). He noted that this is a novel method of solving the NP hard problems. While BBO is a naturally inspired algorithm, it has some fundamental distinctions from common natural algorithms such as genetic algorithm (GA), particle swarm optimization (PSO), or ant colony optimization (ACO). In BBO, the initial population is not discarded among different generations. Instead, the migration concept is used to modify the population. As another distinction, in each generation, the fitness function is not used directly to modify the population, in which BBO uses fitness to determine the immigration and emigration rates. BBO has revealed a good performance on real-world optimization problems such as machining parameters selection [24,25], various power system applications such as economic dispatch [26], optimal power flow [27], and the design placement of phasor measurement units (PMU) [28]. It should be noted that there are no previous studies in the literature which consider using the BBO algorithm to solve the process parameters’ optimization problem for fabricating FSW aluminum joints.

The objective of this research is to model the relationship between the FSW process parameters (tool rotational speed, tool traverse speed, tool tilt angle, tool offset) and the mechanical properties of interest (i.e., tensile strength, minimum hardness in the HAZ zone, and elongation), while a solution algorithm, the multi-objective biogeography based optimization algorithm (MOBBO), is employed to compute the Pareto optimal frontier in objective space. Consequently, the ultimate objective of this study is to obtain the combination of process factors for which the mechanical properties are optimal. The best optimal value of the Pareto frontier for three and two objective functions is selected through two different decision making techniques, TOPSIS and Shannon’s entropy.

## 2. Experimental Works

In this study, dissimilar aluminum alloys (namely AA6061 and AA7075 in T6 temper conditions) were selected to be butt welded by the FSW process. Both plates were of 6 mm thickness and they were cut before welding to a dimension of 100 mm long and 50 mm wide. The weld direction of the FSW is vertical to the rolling direction of the plates. The mechanical properties of the base aluminum alloys, AA6061 and AA7075, are measured before running the experiments. The ultimate tensile strength of AA6061 and AA7075 are 310 MPa and 524 MPa, respectively, and the elongation of AA6061 and AA7075 are 12% and 11%, respectively.

**Figure 1:**
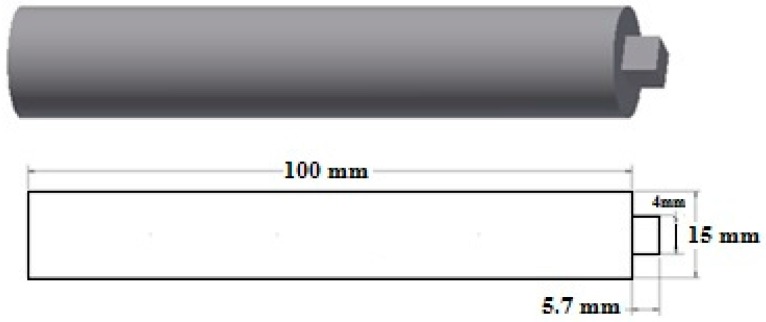
Geometry of the tool design.

A tool made of AISI H13 hot work steel with a square pin profile that was heat treated to a hardness of 52 HRC after machining to increase its wear resistance is used for the experiments (see Figure 1).

The side where the spindle rotation direction is in the same direction with the tool traversing is called the advancing side and the opposite side is called the retreating side. AA6061 was placed in the advancing side and AA7075 was placed in the retreating side in order to improve the mechanical properties of the joint [6,8,29,30].

Based on the literature and previous studies, the parameters with greater influence on the mechanical properties of dissimilar FSW were selected, with their notations and units described in Table 1 [6,31].

Experiments were conducted based on the plan of the surface response, in a central composite design matrix, in which the four factors in five levels were chosen, consisting of 30 sets of coded conditions, which are presented in Table 2. The first 16 experimental runs are derived from the full experimental design matrix of corner points at the ±1 level ($2^{4}=16$). The next eight experimental runs comprise a combination of each process parameter at either the highest (+2) or lowest (−2) level with the other three variables at the middle level (0). The remaining six experimental runs included the variables at the intermediate (0) level constituting the six center points.

**Figure 2:**
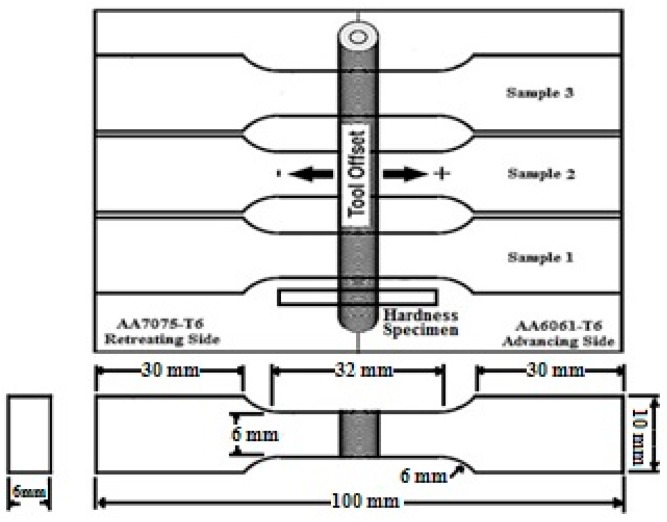
Configuration and dimension of the tensile specimens and tool offset.

Three tensile testing specimens were extracted from the welds. The configuration and dimension of each transverse tensile specimen, tool offset position, and hardness specimen are shown in Figure 2.

Each specimen was conducted according to the ASTM E8M standard at a test speed of 1 mm/min. Care was taken during this stage to align the center of the weld with the center of the tensile specimen. At least three specimens were extracted from each FSWed joint. The tensile testing of the FS welded joints was conducted in a precise Universal Testing Machine Instron 3382, and their UTS and E were measured. To measure the minimum hardness which is located on the AA6061 side in the HAZ regions very close to the thermo-mechanically affected zone (TMAZ) in tensile testing [8], microhardness measurements using a load of 200 g for 20 s were conducted to determine the average minimum hardness of each joint in this region. The average results of the above mentioned tests are presented in Table 2.

Thus the 30 experimental conditions allowed the prediction of the linear, quadratic, and two-way interactive effects of the variables on the ultimate tensile strength (*UTS*), elongation (*E*), and minimum hardness (*H*) of the FS welded joints.

## 3. Developing the Mathematical Models

The mechanical properties *UTS*, *E*, and minimum *H* of the friction stir welded joints are a function of the tool rotational speed (*RS*), traverse speed (*TS*), tool offset (*TO*), and tilt angle (*TA*). They can be expressed as given below

(1) UTS or E or H=f(RS, TS, TO, TA)

The second order polynomial or quadratic polynomial regression equation used to represent the response surface ‘*Y*’ is given by

(2) $$Y=\beta_{0}+\sum \beta_{i}X_{i}+\sum \beta_{ii}X_{i}^{2}+\sum \beta_{ij}X_{i}X_{j}$$

where $\beta_{0}$ is the average value of the responses and $\beta_{i}$, $\beta_{ii}$, and $\beta_{ij}$ are the coefficients of the response which depend on the respective main and interaction effects of the parameters. The second order polynomial regression equation is capable of predicting the accurate mathematical regression models in these kind of problems [14,16,19]. The selected second order polynomial for four factors could be computed as follow

(3) $$\begin{array}{ll} UTS\;\mathrm{or}\;E\;\mathrm{or}\;H= & \beta_{0}+\beta_{1}RS+\beta_{2}TS+\beta_{3}TO+\beta_{4}TA+\beta_{11}RS^{2}+\beta_{22}TS^{2}+\beta_{33}TO^{2}\\ & +\;\beta_{44}TA^{2}+\beta_{12}RS\;TS+\beta_{13}RS\;TO+\beta_{14}RS\;TA+\beta_{23}TS\;TO\\ & +\;\beta_{24}TS\;TA+\beta_{34}TA\;TO \end{array}$$

Response surface analysis of variance (ANOVA) was utilized to predict the combined effect of the rotational speed and the welding process parameters on *UTS*, elongation, and hardness of the dissimilar AA 6061-T6 and AA 7075-T6 alloy welded samples. The least square method was used in regression analysis to find the coefficients of the equation. The values of the coefficients in the polynomial Equations (4)–(6) were computed with the help of the statistical Minitab software. All the response coefficients were tested to predict the mechanical properties of the FSW joint at the 95% confidence level. In order to avoid the computational complexity of the developed model and the cumbersome mathematical labour, the insignificant coefficients of the response were eliminated without affecting the accuracy of the mathematical model. The final developed regression model for predicting *UTS*, *E*, and *H* of the friction stir welded joints with significant control process parameters are given below.

(4) $$UTS\;\left(\mathrm{MPa}\right)=251.667-6.302\;RS+6.33\;TS-2.318\;TA-3.208\;TO-5.271\;RS^{2}-4.771\;TS^{2}-5.347\;TA^{2}-6.558\;TO^{2}$$

(5) $$E\;\left(\%\right)=7.36667-0.6375\;RS+0.54583\;TS-0.55417\;TO-0.22396\;TA^{2}-0.25625\;RS\times TO+0.39375\;TS\times TO$$

(6) $$H\;\left(\mathrm{HV}\right)=70.0833-2.5875\;RS+2.6875\;TS-1.2292\;TO-2.3615\;RS^{2}-2.024\;TS^{2}-3.0615\;TA^{2}-3.2365\;TO^{2}-0.8812\;\mathrm{RS}\times TS+0.8562\;RS\times TO-0.8687\;TS\times TO$$

### 3.1. Checking the Developed Model Accuracy

The statistical results of the mathematical regression models were computed. When the value of $R^{2}$ is equal to one, the predicted values of the responses will ideally match with the corresponding experimental results. The obtained $R^{2}$ value for *UTS*, *E*, and *H* are 0.9462, 0.9382, and 0.9663 respectively, which indicates that the developed models are quite adequate. The developed models’ adequacies were also calculated by analysis of variance (ANOVA) and the statistical results are presented in Table 3.

**Figure 3:**
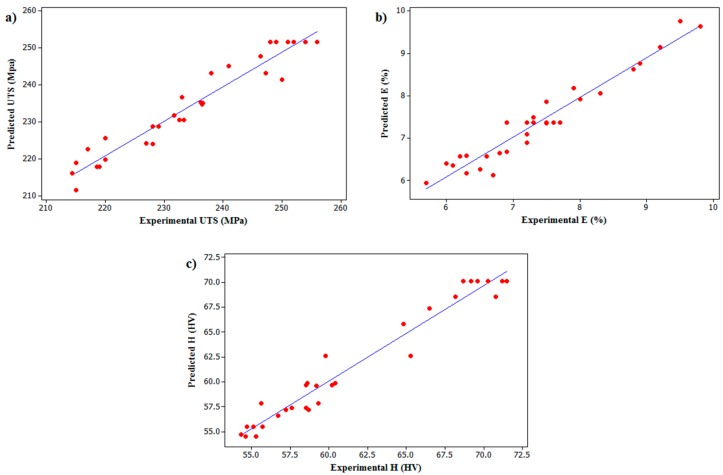
Scatter diagram for: (**a**) *UTS*; (**b**) *E*; and (**c**) *H* of friction stir welded AA6061 and AA7075.

From Table 3, it can be seen that the computed *F*-ratios are greater than that of the corresponding tabulated *F*-ratios at the 95% confidence level, which show that the developed models are adequate. The regression models were developed to predict the *UTS*, *E*, and *H* and plotted scatter diagrams were further employed to validate the models, as shown in Figure 3a–c, respectively. The experimental values and the predicted values obtained from the regression models are scattered at both sides and are close to the 45° line which clearly indicates a good fit of the developed regression models.

### 3.2. Validation of the Regression Models

In order to check the accuracy of the developed models, five different weld runs were made on the AA6061-T6 and AA7075-T6 for different rotational speeds, traverse speeds, tilt angles, and tool offsets other than those used in Table 2. As previously mentioned, three tensile specimens were cut from each friction stir welded joint and their average values of *UTS*, *E*, and *H* were measured. The percentage differences between the predicted and experimental *UTS*, *E*, and *H* were calculated and are presented in Table 4. From this table it is evident that the developed model accuracy for predicting the *UTS*, *E*, and *H* is more than 97.66%, 91.01%, and 94.81%, respectively.

## 4. Multi Objective Optimization

Over the past few years, a number of multi objective evolutionary algorithms have been proposed. The main reason is for the capability to generate multiple Pareto optimal solutions in only one run. The non-dominated sorting genetic algorithm (NSGA) is a widely used non-domination based genetic algorithm used to solve multi-objective optimization problems [32]. The population based search capability in the evolutionary algorithms has made them very suitable for solving multi-objective optimization problems. Thus, employing these algorithms can eliminate the difficulties of classical solution approaches. In the present work, a multi objective biogeography based optimization (MOBBO) algorithm is proposed to deal with the addressed multi-objective process parameter optimization problem. The proposed method consists of two phases: generation of a Pareto frontier by MOBBO, and employing the decision making method to achieve the best compromised optimal solution from the Pareto frontier.

Multi-objective optimization has been defined as an approach to find a vector for all objective functions. Mathematically, the multi-objective optimization process uses a vector of $X^{*}=\left[x_{1}^{*},\;x_{2}^{*},\;\ldots ,\;x_{n}^{*}\right]$ that should be found so as to optimize $F\left(x\right)={\left[f_{1}\left(x\right),f_{2}\left(x\right),\;\ldots ,\;f_{k}\left(x\right)\right]}^{T}$, subject to m inequality constraints, $g_{i}\left(x\right)\leq 0\;\left(i=1\;\mathrm{to}\;m\right)$ and p equality constraints, $h_{i}\left(x\right)=0\;\left(j=1\;\mathrm{to}\;p\right)$ where $X^{*}\in R^{n}$ is the vector of the decision, and $F\left(x\right)\;\in \;R^{k}$ is the vector of objective functions, both of which must be minimized.

In several multi-objective optimizations, the objectives are normally in conflict. Hence, it is impossible to find a solution that satisfies a number of different objectives simultaneously. One of the possible answers for these kinds of problems is a set of solutions, called the Pareto optimal frontier. However, before defining the Pareto optimal frontier, the concept of dominance must be clearly specified. Assume that $x_{1}$ and $x_{2}$ are vectors in *n*-dimensional space and f is a function. $x_{1}$ dominates $x_{2}$ if the following conditions are satisfied:

(7) $$\{\begin{array}{c} f_{i}\left(x_{1}\right)\leq f_{i}\left(x_{2}\right)\;\left(\forall i=1,\ldots ,k\right)\\ \mathrm{and}\\ f_{i}\left(x_{1}\right)<f_{i}\left(x_{2}\right)\;\left(\exists i=1,\ldots ,k\right) \end{array}$$

The Pareto optimal frontier defines a solution that is not dominated by any other points in the solution area. The Pareto optimal solution cannot yield better results regarding an objective unless at least one other objective is worsened. A set of all these non-dominated solutions is called the Pareto optimal frontier. The main goal in multi objective optimization problems is to obtain the Pareto front, which is comprised of Pareto optimum solutions [10].

### 4.1. Biogeography Based Optimization

The BBO is a new evolutionary algorithm among the popular metaheuristic approaches which have arisen as attractive optimization algorithms due to their competitive results [23]. This population-based algorithm is a naturally inspired algorithm which mimics the migration process of species for solving engineering problems [33]. This algorithm has revealed notable performance in many well-known case studies [34]. The BBO algorithm starts the optimization process with a number of candidate solutions, called habitats or islands. Each island feature is considered by a suitability index variable (*SIV*). Each habitat is characterized by a quantitative performance index, named the habitat suitability index (*HSI*). This algorithm starts the optimization process with a number of candidate solutions, named habitats. Each feature of this habitat is considered as a suitability index variable (*SIV*). The performance of each habitat is characterized by an index, called the habitat suitability index (*HSI*), to measure how good the solution is [35].

The main principle of BBO is based on the immigration and emigration of species in a habitat, known as migration. With probabilistic migration, BBO is able to share more information from good solutions to the poor solutions. In other words, this algorithm prevents good solutions from being demolished during its evolution. Thus, it can efficiently utilize the characteristics and information of the population per iteration. This feature leads to finding better solutions in a short time as opposed to other metaheuristics [36]. In evolutionary strategies, the recombination approach is employed to generate a new solution, while in BBO the migration operator is executed to make changes within existing solutions. Recombination in evolutionary algorithms is a reproductive process, whereas the migration operator in BBO is an adaptive process, which is employed to alter an existing population [37].

Additionally, the mutation operator increases the diversity among the population. Without the mutation operator that can increase diversity among the population, the solutions with high HSI have a tendency to be more dominant in the population. The mutation approach makes both solutions with low or high HSI likely to mutate and gives a chance of improvement to both types of solutions in comparison with their earlier values [38].

#### 4.1.1. Initialize the Habitats

Defining an appropriate habitat representation plays a key role in designing and implementing an optimization algorithm. Each habitat is a potential solution to the problem. The habitat length depends on the number of decision variables. To solve the addressed optimization problem, a real-valued habitat representation is employed in which the variables, the process parameters, are randomly generated within its minimum and maximum bounds. The individual habitat is represented as $\left[SIV_{1},\;SIV_{2},\;\ldots ,\;SIV_{N}\right]$ in which N denotes the number of variables. The objective function or *HIS* of the habitats can be computed.

#### 4.1.2. Migration

In biogeography, migration is the adaptive process which is used to move the species between different habitats. The migration process occurs based on a probabilistic operator. In BBO, the probability of selecting the solution $H_{i}$ as the immigrating habitat is related to its immigration rate $\lambda_{i}$ and the solution $H_{j}$ is related to its emigration rate$\;\mu_{i}$. Migration can be express as Equation (8).

(8) $$H_{i}\left(SIV\right)⟵H_{j}\left(SIV\right)$$

The immigration and emigration rates are functions of the solutions’ fitness. They can be evaluated by Equations (9) and (10), respectively.

(9) $$\lambda_{i}=I\left(1-\frac{k_{i}}{n}\right)$$

(10) $$\mu_{i}=E\left(\frac{k_{i}}{n}\right)$$

In Equations (9) and (10), I and E represent the maximum possible immigration and emigration rate, respectively; $k_{i}$ is the rank of habitat i after sorting all habitats according to their HSI; and n is the number of solutions in the population. It is clear that the better solution has a higher emigration and a lower immigration rate, while the opposite is true for a poor solution. Often, I and E set equal to one or slightly less than one [39].

After determining the immigrating and emigrating habitats, the migration process can be performed like a crossover in evolutionary algorithms. In this study, the migration process for each habitat vector is implemented separately.

To perform the migration, an arithmetic crossover operator that defines a linear combination of two chromosomes is used. The selected immigrating and emigrating habitats may produce two new habitats, called modified habitats, by taking the linear combination of the habitats’ SIVs as follows:

$${\mathrm{Modified}\;\mathrm{habitat}}_{1}=\alpha \times \mathrm{Immigrating}\;\mathrm{habitat}+\left(1-\alpha \right)\times \mathrm{Emigrating}\;\mathrm{habitat}$$

$${\mathrm{Modified}\;\mathrm{habitat}}_{2}=\left(1-\alpha \right)\times \mathrm{Immigrating}\;\mathrm{habitat}+\alpha \times \mathrm{Emigrating}\;\mathrm{habitat}$$

where α is the weight which governs the dominant individual in reproduction and it is between 0 and 1.

#### 4.1.3. Mutation

In BBO, the mutation rate is inversely related to the solution probability and can be calculated by Equation (11).

(11) $$m_{i}=m_{\max}\times \left(1-\frac{P_{s}}{P_{\max}}\right)$$

In Equation (11), $m_{\max\;}$ is a user-defined maximum probability, $P_{\max}=\mathrm{argmax}\;P_{s},\;S=1,2,\ldots ,S_{\max}$ ($S_{\max}$ is the population size), and $P_{s}$. is the solution probability. In the mathematics of biogeography, the solution probability depends on the immigration and emigration rate of the habitat and can be calculated by Equation (12).

(12) $$P_{s}=\{\begin{array}{cc} -(\lambda_{s}+\mu_{s})P_{s}+\mu_{s+1}\;P_{s+1} & S=0\\ -(\lambda_{s}+\mu_{s})P_{s}+\lambda_{s-1}\;P_{s-1}+\mu_{s+1}\;P_{s+1} & 1\leq S\leq S_{\max}\\ -(\lambda_{s}+\mu_{s})P_{s}+\lambda_{s-1}\;P_{s-1} & S=S_{\max} \end{array}$$

The species count in the habitat changes from time to time. $\lambda_{s}$ and $\lambda_{s-1}$ are the immigration rates of the habitat having S and S−1 species, respectively. $\mu_{s}$ and $\mu_{s+1}$ are the emigration rates of the habitat having S and S+1 species, respectively. $P_{s}$, $P_{s+1}$, and $P_{s-1}$ are the species count probabilities of the habitat with S, S+1, and S−1 species, respectively. $S_{\max}$ is the maximum species count in the habitat [23].

Based on the mutation probability, the mutation operators can be implemented to make a random change in the *m*th *SIV* selected randomly for the habitat by generating a random real value within its minimum and maximum limits.

#### 4.1.4. Evaluating the *HSI*

In BBO, each habitat is evaluated based on its corresponding *HSI*. Generally, the fitness value is measured according to the objective function.

#### 4.1.5. Update Habitat

To update the population for the next generation in single objective optimization, three steps (merging, sorting, and truncating) have been implemented. This scheme is used to preserve elite habitats for the next generation. Merging is related to the combination of habitats, before and after applying BBO operators which makes the habitat population size double at a time. Then, the combined habitats must be sorted based on their *HSI* in ascending order (in the minimization problem). Finally, the best habitats are selected from the combined and sorted habitat with the amount of the original habitat size for the next generation.

In multi-objective biogeography based optimization, the habitats are updated based on the non-dominated sorting and ranking (NDSR) scheme proposed by [32]. This scheme is employed to determine the Pareto optimal frontier. The newly generated habitats are combined with the habitats before and after executing the BBO operators, which makes the original habitat size double at a time. Then, the NDSR procedure is performed on the updated and combined habitats. A diversity rank is allocated to the solution vectors which are in the same non-dominated front, using the crowding distance metric.

This metric is an indication of the density of the solution vectors neighbouring a particular solution vector. The crowding distance can be measured for the *k*th objective of the *j*th solution as follows.

(13) $$d_{j,k}=\frac{F_{k,j-1}-F_{k,j+1}}{F_{k,\max}-F_{k,\min}}$$

An infinite distance index is assigned to the solutions having the smallest and largest values. The overall crowding distance is measured by using the sum of the individual distance values of each objective.

(14) $$D_{j}=\overset{m}{\underset{k=1}{\sum }}d_{j,k}$$

where m gives the number of objectives and j is the individual index. Finally, the best habitat is selected from the combined habitat in the order of their ranking for the next iteration.

#### 4.1.6. Stopping Criterion

The optimization algorithm with various operators such as migration and mutation is performed repeatedly until a stopping criterion is met. In this study, the BBO algorithm is stopped when the number of generations has been met.

#### 4.1.7. Implementation

Based on aforementioned BBO operators, the BBO algorithm used to solve the scheduling problem in FMS is described as follows.

Generate a set of habitats for a problemEvaluate the fitness value or *HSI* for each habitatwhile stopping criterion is not met Determine immigrating rate λ and emigrating rate μ for each habitatModify the habitats based on λ and μfor i=1: *N* (population size) doUse λ to probabilistically decide whether to modify to emigrationif rand (0, 1) < $\lambda_{i}$Select Habitat $H_{j}$ through roulette wheel method to emigrationPerform migration on $H_{i}$ and $H_{j}$Evaluate the fitness value or *his*Replace the new solution with $H_{i}$end ifif rand (0, 1) < $P_{Mutation}$Apply mutation on $H_{i}$Evaluate the fitness value or *HSI* for the newly generated solutionend ifend forUpdate habitats’ populationend while

### 4.2. Objective Function, Decision Variables, and Constraints

This study has been carried out using the ultimate tensile strength, yield strength, elongation, and hardness function as the four objective functions, as given in Equations (4)–(6) with the tool rotational speed (*RS*), tool traverse speed (*TS*), tilt angle (*TA*), and tool offset (*TO*) as the four decision variables. The objective function and constraints for carrying out the multi objective optimization are expressed as follows.

Max: ultimate tensile strength (RS,TS,TA,TO)
Max: elongation (RS,TS,TA,TO)
Max: hardness (RS,TS,TA,TO)
Subject to:
  800≤rotational speed≤1600
  20≤traverse speed≤180
  1°≤tilt angle≤3°
  −2≤tool offset≤2

### 4.3. Decision Making Methods in Multi-Objective Optimization

The Pareto set has numerous optimal solutions which are preferred based on the decision making requirements. Hence, the decision making methods are important to pick the best solution from the Pareto frontier. Several multi criteria decision making (MCDM) methods are proposed in the literature [40,41]. This study considers two decision making approaches, TOPSIS and Shannon’s entropy. Few decision makers evaluate a concrete solution on the basis of how far the solution is from nadir, while others use the proximity index with the ideal solution. The decision making methods used in this study are described in the following section.

#### 4.3.1. Shannon Entropy Method

Shannon’s entropy approach is a useful technique to determine the hold weights of alternatives [42]. This approach gives a matrix $L_{ij}$ with a decision matrix $F_{ij}$ considering m different alternatives with n objectives in the system. For the *j*th objective, arrays of the $L_{ij}$ matrix can be computed for the formulae:

(15) $$L_{ij}=\frac{F_{ij}}{\sum_{i=1}^{m}F_{ij}}\;,\;i=1,\;2,\;\ldots ,\;m,\;j=1,\;2,\;\ldots ,\;n$$

Shannon’s entropy is presented as:

(16) $$SE_{j}=-M\;\overset{n}{\underset{i=1}{\sum }}L_{ij}\ln L_{ij},\;M=1/\ln\left(n\right)$$

The deviation ($D_{j}$) degree is defined as:

(17) $$D_{j}=1-\;SE_{j}$$

The following equation calculates the *j*th objective weights as:

(18) $$W_{j}=\frac{D_{j}}{\sum_{j=1}^{n}D_{j}}$$

To end with $Y_{i}=L_{ij}\;W_{j}$, Shannon’s entropy method finds the point on the Pareto optimal frontier where the maximum $Y_{i}$ is the final desired optimal result. Therefore,

(19) $$i_{final}\equiv i\in \max(Y_{i})\;i=1,\;2,\;\ldots ,\;m$$

#### 4.3.2. TOPSIS Decision Making Method

TOPSIS is an MCDM approach which is used to rank the alternatives of the obtained Pareto solutions. The basic concept of TOPSIS is to obtain the best compromise solution according to the objective weights. It is clear that in the multi-objective optimization problem it is impossible to find the optimal condition of each objective achieved by a single objective optimization. Thus, the ideal solution and non-ideal solution are not located on the Pareto frontier. The ideal (non-ideal) point is the coordination in the objective’s solution area in which each objective has its best (worst) value. In order to select the best compromise solution, an evaluation matrix ${\left(F_{ij}\right)}_{m\times n}$ should be created (*m* alternatives and *n* criteria). $F_{ij}$ is also the *j*th objective value of the *i*th alternative. The computed matrix should then be non-dimensionalized through the Euclidian approach using Equation (20):

(20) $$L_{ij}=\frac{F_{ij}}{\sqrt{\sum_{i=1}^{m}{\left(F_{ij}\right)}^{2}}}$$

The weighted non-dimensionalized decision matrix is computed as follows:

(21) $$Y_{ij}={\left(L_{ij}\;W_{j}\right)}_{m\times n}$$

where $W_{j}$ is the weight calculated by Shannon’s entropy method. The solution distance on the Pareto optimal frontier from the ideal point denoted by $d_{i+}$ is measured as follows:

(22) $$d_{i+}=\sqrt[{2}]{\sum_{j=1}^{n}{\left(Y_{ij}-Y_{j}^{ideal}\right)}^{2}}$$

where n denotes the number of objectives while i represents each solution on the Pareto frontier (i=1, 2, …, m). In Equation (22), $Y_{j}^{ideal}$ is the ideal value for the *j*th objective. In addition to the distance of each solution from the ideal point $d_{i+}$, the solution distance from the non-ideal point denoted by $d_{i-}$ should be calculated. Therefore,

(23) $$d_{i-}=\sqrt[{2}]{\sum_{j=1}^{n}{\left(Y_{ij}-Y_{j}^{non-ideal}\right)}^{2}}$$

In computing the TOPSIS method, the $CI_{i}$ parameter is defined as follows:

(24) $$CI_{i}=\frac{d_{i-}}{d_{i+}-d_{i-}}$$

In the TOPSIS approach, a solution with a maximum $CI_{i}$ is chosen as a desired ultimate solution, therefore, $i_{final}$ is the index for the final selected solution, which is:

(25) $$i_{final}\equiv i\in \max(CI_{i})\;i=1,\;2,\;\ldots ,\;m$$

## 5. Results and Discussion

The optimal value of the ultimate tensile strength, elongation, and hardness function is obtained by implementing the evolutionary algorithm based on the multi objective BBO algorithm. The optimization problem is implemented by the proposed MOBBO algorithm coded using Matlab R2013a and run on an Intel^®^ core™ CPU i7-4500U at 1.8 GHZ, 8 GB RAM computer with Windows 8.1. The MOBBO parameters are set in this study after a number of careful runs as follows; the habitat size (N) = 70, maximum migration and immigration rate of each habitat = 1, mutation probability = 0.05, and maximum iteration = 300.

The final optimal solution selected by the TOPSIS and Shannon’s entropy decision making approaches for the triple objective and the results of two and single objective were computed. As can be seen in Table 5, the ideal and nadir solution for the three objectives’ (*UTS-E-H*) optimization are 256.28 MPa, 10.92%, 72.62 HV, and 216.93 MPa, 7.13%, 63.02 HV, respectively. Therefore, the optimal solution for the triple objective optimization lies in 216.93 MPa≤UTS≤256.28 MPa, 7.13%≤E≤10.92%, and 63.02 HV≤H≤72.62 HV. It is clearly seen that the optimal solution selected by the decision making methods for the triple objective optimization is inclined towards a rotational speed from 967.41 to 1002.41 rpm, traversing speed from 164.40 to 149.73 mm/min, tilt angle from 1.92° to 1.97°, and tool offset from −0.74 to −1.05 mm.

**Figure 4:**
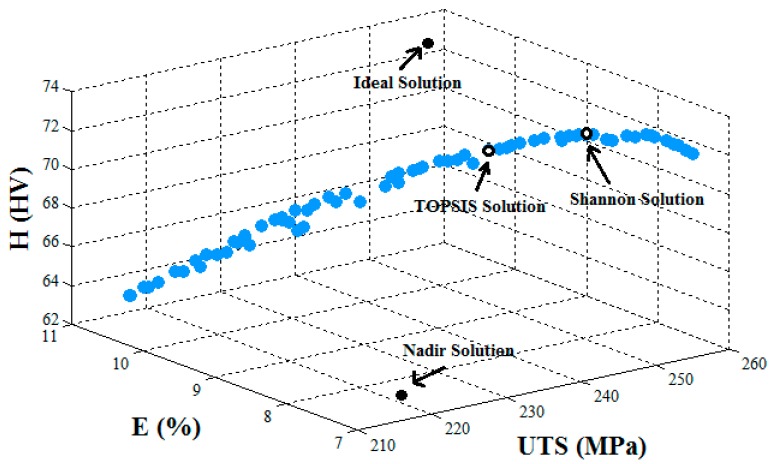
Pareto frontier for the triple objective (*UTS-E-H*).

Figure 4 shows the Pareto frontier for the triple objective (*UTS-E-H*) with the best optimal solutions selected through the TOPSIS and Shannon’s entropy decision making approaches, as well as the ideal and nadir solutions.

In order to measure the distinction between the results obtained, the deviation index of the results obtained by two different decision making approaches from the ideal value is computed as follows:

(26) $$D=\frac{d_{1}}{d_{1}+d_{2}}\;\mathrm{where},$$

(27) $$d_{1}=\sqrt{\sum_{i=1}^{n}{\left(F_{i}-F_{i}^{ideal}\right)}^{2}}\;\mathrm{and}\;d_{2}=\sqrt{\sum_{i=1}^{n}{\left(F_{i}-F_{i}^{nadir}\right)}^{2}}$$

The deviation index of the optimal solution obtained by TOPSIS and Shannon’s entropy for the triple and dual optimization approaches are illustrated in bold in the last column of Table 5. Shannon’s entropy decision making method yields a lower value of the deviation index as 0.12 with the triple objective optimization.

**Figure 5:**
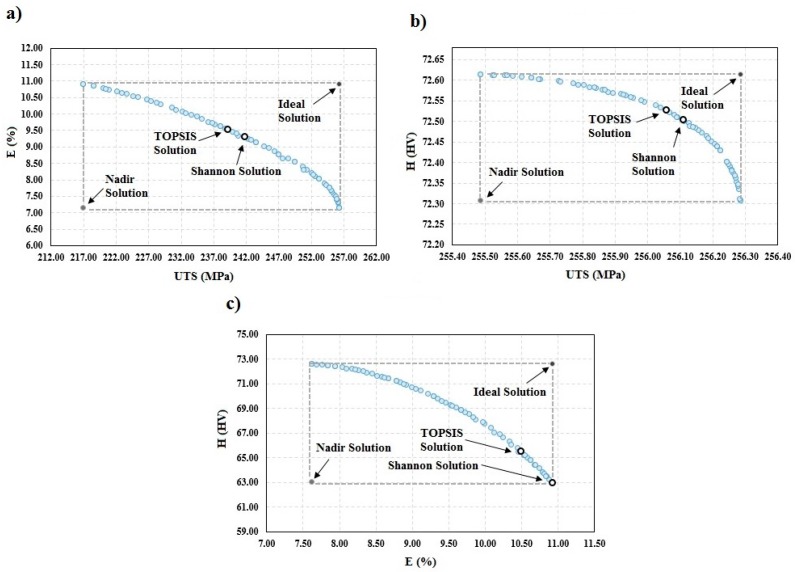
Pareto frontier for: (**a**) dual objective (*UTS-E*); (**b**) dual objective (*UTS-H*); and (**c**) dual objective (*E-H*) optimization.

Figure 5a shows the optimal results for the dual objective (*UTS-E*) optimization. The optimal results based on the two decision making methods and the ideal and non-ideal solutions are indicated in this figure. The optimal solution’s population in 216.93 MPa≤UTS≤256.29 MPa and 7.15%≤E≤10.92% can be seen in this figure. Therefore, the optimal answers are probably in this region and as it is mentioned, the results of TOPSIS and Shannon’s entropy are in this region. As shown in Table 5, the chosen responses through Shannon’s entropy method are the closest to the ideal solution with a deviation index of 0.37. Similarly, the optimal results for the other dual objective optimization (*UTS-H* and *E-H*) are illustrated in Figure 5b,c, respectively, and the obtained solutions through the decision making methods are displayed.

The final optimal solution of the dual objective optimization picked from the Pareto frontier by the decision making methods with the lowest value of the deviation index are shown in bold font in the last column of Table 5.

It is observed from Figure 5a,c that the elongation percentage function dramatically decreases with the increase in the ultimate tensile strength and minimum hardness in the HAZ area. Hence, with the use of the obtained Pareto frontier, one has to determine the point of maximum overall elongation without considerable compromise in the ultimate tensile strength and minimum hardness. The final optimal solution for the single optimization is illustrated in Table 5 for comparison purposes.

**Figure 6:**
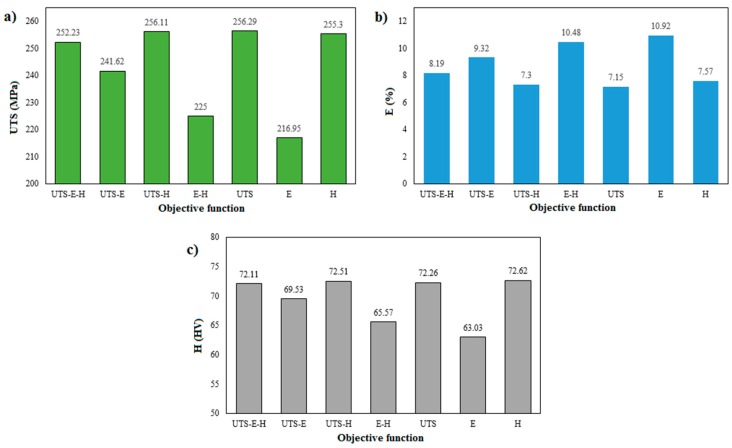
Comparison of: (**a**) *UTS*; (**b**) *E*; and (**c**) *H* obtained through various optimizations.

Figure 6 shows comparative studies of the ultimate tensile strength, elongation, and minimum hardness in the HAZ zone function obtained through various optimization schemes. Therefore, it is concluded that three objective optimization can lead to more desired results as compared to dual and single objective optimization.

It is evident that considering all of the competing criteria such as *UTS*, *E*, and *H* in optimizing the process parameters of dissimilar FSW welded AA6061-T6 and AA7075-T6 joints is impossible without solving a multi-objective optimization procedure. However, it should be noted that there are no stringent protocols for the application of decision making methods, as other techniques might produce superior results for the same problem.

In order to carry out error analysis of the implemented decision making methods used in this study, mean absolute percentage error (MAPE) is applied. To achieve this, 30 different runs for the computer code were performed and a final solution for each run was obtained using the Shannon’s entropy and TOPSIS decision making methods. Then, the magnitudes of each objective (ultimate tensile strength, elongation, and minimum hardness in the HAZ zone) were compared with the best solutions gained after 30 runs by each method. The first three columns of Table 6 demonstrate the average percentage error and second three columns display the maximum percentage error of the decision making methods for each objective.

The MAPE technique has been executed for the error analysis which shows that the average error and maximum error of the solutions attained from the two decision making methods are 0.41%, 2.22%, 0.24% and 0.85%, 4.13%, 0.59% for the ultimate tensile strength, elongation, and minimum hardness in the HAZ zone, respectively. According to the results, the Shannon approach has a better solution for *UTS*, *E*, and *H* in contrast with other decision making methods.

## 6. Conclusions

Friction stir welding with different process parameters was developed successfully by using a conventional milling machine for dissimilar aluminum alloys AA6061-T6 and AA7075-T6. The mathematical regression model was developed based on an experimental study on ultimate tensile strength, elongation, and minimum hardness of the dissimilar FS welded joints, and was validated. Analysis of the experimental data by the developed model revealed that the mathematical regression model can be employed to predict the aforementioned mechanical properties of the dissimilar friction stir welded joints of AA6061 and AA7075 Al alloys within ±9% of their experimental values at a 95% confidence level.

In order to deal with this multi response optimization problem, a multi objective optimization algorithm was proposed. This proposed solution algorithm is comprised of two phases: generation of a Pareto set by MOBBO, and the use of two different decision making methods (Shannon’s entropy and TOPSIS) to achieve the best compromise solution from the Pareto set.

The results indicated that the multi-objective BBO algorithm in combination with the mathematical regression model is a useful technique to optimize the FSW process parameters to obtain the maximum mechanical properties of the FSW joints. The best theoretical compromise solution chosen by Shannon’s entropy for the three-objective optimization has the optimal values for the tool rotational speed of 1002.14 rpm, tool traversing speed of 149.73 mm/min, tilt angle of 1.92°, and tool offset of −0.74 mm at which the ultimate tensile strength, elongation, and hardness are 252.23 MPa, 8.19%, and 72.11 HV, respectively. By setting those parameters as close as possible to the theoretical values in the vertical milling machine, the multi-objective optimum experimental values of *UTS*, *E*, and *H* are 253 MPa, 8.2%, and 71.4 HV, respectively. Further research can be carried out by combining multi-objective biogeography based optimization with other decision making methods such as fuzzy bellman Zadeh or linear programming technique for multidimensional analysis of preference (LINMAP) to obtain the best compromise solution from the Pareto set.

## Figures and Tables

**Table 1:** Process parameters and their levels.

No.	Parameters	Notation	Unit	Levels				
				−2	−1	0	+1	+2
1	Rotational speed	*RS*	rpm	800	1000	1200	1400	1600
2	Traverse speed	*TS*	mm/min	20	60	100	140	180
3	Tool offset	*TO*	mm	−2	−1	0	1	2
4	Tilt angle	*TA*	(°)	1	1.5	2	2.5	3

**Table 2:** Design matrix and experimental results.

Test Run	Process Parameters				Experimental Values		
	RS (rpm)	TS (mm/min)	TA (°)	TO (mm)	UTS (MPa)	E (%)	H (HV)
R01	−1	−1	−1	−1	236.56	8.3	60.2
R02	1	−1	−1	−1	217	5.7	55.3
R03	−1	1	−1	−1	246.32	9.5	68.2
R04	1	1	−1	−1	236.25	8	60.4
R05	−1	−1	1	−1	232.58	7.5	58.5
R06	1	−1	1	−1	218.6	6.7	54.6
R07	−1	1	1	−1	247.26	9.8	70.8
R08	1	1	1	−1	233.33	7.9	58.6
R09	−1	−1	−1	1	229	7.5	57.2
R10	1	−1	−1	1	214.4	6.5	54.7
R11	−1	1	−1	1	250	7.3	65.3
R12	1	1	−1	1	228	6.9	58.5
R13	−1	−1	1	1	228	7.2	58.7
R14	1	−1	1	1	215	6.3	55.1
R15	−1	1	1	1	233	7.2	59.8
R16	1	1	1	1	226.9	6.8	57.6
R17	2	0	0	0	219	6.3	55.7
R18	−2	0	0	0	238	9.2	64.8
R19	0	2	0	0	241	8.9	66.5
R20	0	−2	0	0	220	6.2	56.7
R21	0	0	2	0	220	6.1	55.6
R22	0	0	−2	0	236.39	6.6	59.3
R23	0	0	0	2	215	6	54.3
R24	0	0	0	−2	231.7	8.8	59.2
R25	0	0	0	0	248	7.5	71.5
R26	0	0	0	0	249	7.7	68.7
R27	0	0	0	0	254	7.2	69.2
R28	0	0	0	0	256	7.3	71.2
R29	0	0	0	0	252	6.9	69.6
R30	0	0	0	0	251	7.6	70.3

**Table 3:** ANOVA results for the developed regression models.

Responses	Source	Sum-of-Square	DF	Mean-Square	*F*-Ratio (Calculated)	*F*-Ratio (Tabulated)	*p*-Value
*UTS*	Regression	4720.48	8	337.18	18.83	2.42	0.000
	Residual	268.58	21	17.91	-	-	-
*E*	Regression	30.52	6	2.18	16.27	2.53	0.000
	Residual	2.01	23	0.134	-	-	-
*H*	Regression	995.68	10	71.12	30.74	2.38	0.000
	Residual	34.71	19	2.31	-	-	-

DF: degrees of freedom; *F*-ratio: mean sum-of-squares for regression/mean sum-of-squares for residual *p*-value: the smallest level of significance at which the data are significant.

**Table 4:** Results of the conformity tests for the developed models of *UTS*, *E*, and *H*.

No	Parameters				Experimental Value			Predicted Value			% of Error		
	*RS*	*TS*	*TA*	*TO*	*UTS*	*E*	*H*	*UTS*	*E*	*H*	*UTS*	*E*	*H*
1	0.5	1.25	−0.25	−1	241.2	8.5	64.8	244.6	8.6	66.3	1.37	1.56	2.27
2	−1	0.75	−0.25	0.5	253.6	8.1	70.2	251.8	7.8	69.5	0.73	3.23	1.04
3	0.75	−0.25	0.5	−1	232.7	7.2	59.3	236.2	7.0	62.5	1.50	3.45	5.19
4	−2	1.25	−1.5	−1.25	234.2	9.8	60.2	228.9	10.8	61.3	2.34	8.99	1.80
5	0.25	−0.75	1.5	0	231.2	6.5	58.8	226.8	6.3	59.4	1.93	3.27	1.03

% of Error = [(experimental value − predicted value)/predicted value] × 100.

**Table 5:** The values of the design variables and objective functions of the optimum points specified using the decision making approach.

Objective Functions	Solution Methods	Process Parameters				Mechanical Properties			Deviation Index
		RS (rpm)	TS (mm/min)	TA (°)	TO (mm)	UTS (MPa)	E (%)	H (HV)	
*UTS-E-H*	TOPSIS	967.41	164.40	1.97	−1.05	245.95	8.92	70.85	0.26
	Shannon	1002.14	149.73	1.92	−0.74	252.23	8.19	72.11	0.12
	Ideal	-	-	-	-	256.28	10.92	72.62	0
	Nadir	-	-	-	-	216.93	7.13	63.02	1
*UTS-E*	TOPSIS	977.07	174.67	1.97	−1.40	239.07	9.54	68.79	0.44
	Shannon	986.39	174.43	1.96	−1.25	241.62	9.32	69.53	0.37
	Ideal	-	-	-	-	256.29	10.92	-	0
	Nadir	-	-	-	-	216.93	7.15	-	1
*UTS-H*	TOPSIS	1064.69	131.52	1.96	−0.32	256.06	7.33	72.53	0.28
	Shannon	1066.10	130.53	1.96	−0.31	256.11	7.30	72.51	0.24
	Ideal	-	-	-	-	256.28	-	72.62	0
	Nadir	-	-	-	-	255.49	-	72.31	1
*E-H*	TOPSIS	831.43	180.00	2.00	−1.72	225.00	10.48	65.57	0.65
	Shannon	800.00	180.00	2.00	−2.00	216.95	10.92	63.03	0.74
	Ideal	-	-	-	-	-	10.92	72.62	0
	Nadir	-	-	-	-	-	7.61	63.03	1
*UTS*	-	1080.44	126.54	1.89	−0.24	256.29	7.15	72.26	-
*E*	-	800.00	180.00	2.00	−2.00	216.95	10.92	63.03	-
*H*	-	1040.61	137.10	2.00	−0.42	255.30	7.57	72.62	-

**Table 6:** Error analysis based on the mean absolute percentage error (MAPE) method.

Decision Making Methods	Average Percentage Error			Max Percentage Error		
	UTS (MPa)	E (%)	H (HV)	UTS (MPa)	E (%)	H (HV)
TOPSIS	0.41	2.22	0.24	0.85	4.13	0.59
Shannon	0.11	1.68	0.03	0.31	3.10	0.22
